# A 10-Year Longitudinal Study of Brain Cortical Thickness in People with First-Episode Psychosis Using Normative Models

**DOI:** 10.1093/schbul/sbae107

**Published:** 2024-07-05

**Authors:** Pierre Berthet, Beathe C Haatveit, Rikka Kjelkenes, Amanda Worker, Seyed Mostafa Kia, Thomas Wolfers, Saige Rutherford, Dag Alnaes, Richard Dinga, Mads L Pedersen, Andreas Dahl, Sara Fernandez-Cabello, Paola Dazzan, Ingrid Agartz, Ragnar Nesvåg, Torill Ueland, Ole A Andreassen, Carmen Simonsen, Lars T Westlye, Ingrid Melle, Andre Marquand

**Affiliations:** Department of Psychology, University of Oslo, Oslo, Norway; Norwegian Center for Mental Disorders Research (NORMENT), University of Oslo, and Oslo University Hospital, Oslo, Norway; Department of Psychology, University of Oslo, Oslo, Norway; Norwegian Center for Mental Disorders Research (NORMENT), University of Oslo, and Oslo University Hospital, Oslo, Norway; Department of Psychology, University of Oslo, Oslo, Norway; Norwegian Center for Mental Disorders Research (NORMENT), University of Oslo, and Oslo University Hospital, Oslo, Norway; Department of Psychosis Studies, Institute of Psychiatry, King’s College, London, UK; Donders Institute for Brain, Cognition, and Behaviour, Radboud University, Nijmegen, the Netherlands; Department of Psychiatry, Utrecht University Medical Center, Utrecht, the Netherlands; Department Cognitive Science and Artificial Intelligence, Tilburg University, the Netherlands; Department of Psychology, University of Oslo, Oslo, Norway; Norwegian Center for Mental Disorders Research (NORMENT), University of Oslo, and Oslo University Hospital, Oslo, Norway; Department of Psychiatry and Psychotherapy, Tübingen Center for Mental Health, University of Tübingen, Tübingen, Germany; Donders Institute for Brain, Cognition, and Behaviour, Radboud University, Nijmegen, the Netherlands; Department of Cognitive Neuroscience, Radboud University Medical Center, Nijmegen, the Netherlands; Department of Psychiatry, University of Michigan, Ann Arbor, MI, USA; Department of Psychology, University of Oslo, Oslo, Norway; Norwegian Center for Mental Disorders Research (NORMENT), University of Oslo, and Oslo University Hospital, Oslo, Norway; Department Cognitive Science and Artificial Intelligence, Tilburg University, the Netherlands; Department of Psychology, University of Oslo, Oslo, Norway; Norwegian Center for Mental Disorders Research (NORMENT), University of Oslo, and Oslo University Hospital, Oslo, Norway; Department of Psychology, University of Oslo, Oslo, Norway; Norwegian Center for Mental Disorders Research (NORMENT), University of Oslo, and Oslo University Hospital, Oslo, Norway; Department of Psychology, University of Oslo, Oslo, Norway; Norwegian Center for Mental Disorders Research (NORMENT), University of Oslo, and Oslo University Hospital, Oslo, Norway; Department of Psychosis Studies, Institute of Psychiatry, King’s College, London, UK; Norwegian Center for Mental Disorders Research (NORMENT), University of Oslo, and Oslo University Hospital, Oslo, Norway; Department of Psychiatric Research, Diakonhjemmet Hospital, Oslo, Norway; Centre for Psychiatry Research, Department of Clinical Neuroscience, Karolinska Institutet, Stockholm, Sweden; Department of Mental Disorders, Norwegian Institute of Public Health, Oslo, Norway; Department of Psychology, University of Oslo, Oslo, Norway; Norwegian Center for Mental Disorders Research (NORMENT), University of Oslo, and Oslo University Hospital, Oslo, Norway; Norwegian Center for Mental Disorders Research (NORMENT), University of Oslo, and Oslo University Hospital, Oslo, Norway; Norwegian Center for Mental Disorders Research (NORMENT), University of Oslo, and Oslo University Hospital, Oslo, Norway; Department of Psychiatric Research, Diakonhjemmet Hospital, Oslo, Norway; Department of Psychology, University of Oslo, Oslo, Norway; Norwegian Center for Mental Disorders Research (NORMENT), University of Oslo, and Oslo University Hospital, Oslo, Norway; Norwegian Center for Mental Disorders Research (NORMENT), University of Oslo, and Oslo University Hospital, Oslo, Norway; Donders Institute for Brain, Cognition, and Behaviour, Radboud University, Nijmegen, the Netherlands; Department of Cognitive Neuroscience, Radboud University Medical Center, Nijmegen, the Netherlands

**Keywords:** schizophrenia, cortical thickness, long-term follow up, normative modeling

## Abstract

**Background:**

Clinical forecasting models have potential to optimize treatment and improve outcomes in psychosis, but predicting long-term outcomes is challenging and long-term follow-up data are scarce. In this 10-year longitudinal study, we aimed to characterize the temporal evolution of cortical correlates of psychosis and their associations with symptoms.

**Design:**

Structural magnetic resonance imaging (MRI) from people with first-episode psychosis and controls (*n* = 79 and 218) were obtained at enrollment, after 12 months (*n* = 67 and 197), and 10 years (*n* = 23 and 77), within the Thematically Organized Psychosis (TOP) study. Normative models for cortical thickness estimated on public MRI datasets (*n* = 42 983) were applied to TOP data to obtain deviation scores for each region and timepoint. Positive and Negative Syndrome Scale (PANSS) scores were acquired at each timepoint along with registry data. Linear mixed effects models assessed effects of diagnosis, time, and their interactions on cortical deviations plus associations with symptoms.

**Results:**

LMEs revealed conditional main effects of diagnosis and time × diagnosis interactions in a distributed cortical network, where negative deviations in patients attenuate over time. In patients, symptoms also attenuate over time. LMEs revealed effects of anterior cingulate on PANSS total, and insular and orbitofrontal regions on PANSS negative scores.

**Conclusions:**

This long-term longitudinal study revealed a distributed pattern of cortical differences which attenuated over time together with a reduction in symptoms. These findings are not in line with a simple neurodegenerative account of schizophrenia, and deviations from normative models offer a promising avenue to develop biomarkers to track clinical trajectories over time.

## Introduction

Psychotic disorders are severe and complex conditions characterized by substantial clinical and biological heterogeneity^1–3^ and significant negative effects on quality of life and societies.^4–7^ Predicting long-term outcomes and improving treatment and prognosis are a priority in schizophrenia research, and models that can predict the clinical course are highly needed, as this will help us to optimize treatment planning. Longitudinal clinical studies have revealed substantial heterogeneity in the clinical and functional trajectories,^8–12^ underlying neurobiology,^13–16^ and its interaction with medication.^17–21^ Likewise, both cross-sectional and longitudinal brain imaging studies have revealed significant yet typically diffuse brain cortical alterations in groups of individuals with psychotic disorders.^22–24^ Both neurodevelopmental and neurodegenerative models have been proposed for the evolution of these changes,^25–27^ and there is increasing evidence and awareness of substantial individual differences and heterogeneity in such trajectories.^28–30^ However, the timeframe for most longitudinal studies is relatively short (e.g., 1–2 years) and there is a need for better characterization of the dynamics of the brain cortical correlates of the illness and their long-term temporal associations with clinical symptoms at an individual level.^31–35^ Two previous long-term prospective studies of first-episode psychosis patients were conducted more than 20 years ago.^36,37^ These studies comprised patients receiving first-generation antipsychotics, a group of medications associated with findings of reduced cortical thickness (CT).^18,38^ A more recent study reports an association between increasing expressive negative symptoms and changes in CT, reporting on the dose but not the type of medication.^39^

More recently still, the availability of large neuroimaging datasets has led to the advent of normative development charts^40,41^ that allow for individual-level statistical inference and for mapping clinical traits to extreme deviations from the normative range.^28,42–49^ Such techniques may be particularly valuable in longitudinal studies because they provide the ability to detect deviations from an expected trajectory over time, which might provide early indicators of worsening or improvement in the disease course and can accommodate heterogeneity in the pattern of atypicalities across individuals and timepoints.

Our main goal in this study was to map the associations between brain cortical abnormalities and clinical symptoms over the longer term. To achieve this, we applied a normative modeling approach to magnetic resonance imaging (MRI)-based estimates of cerebral CT of people with schizophrenia spectrum first-episode psychosis and healthy controls (CTRL) in a long-term longitudinal study of participants with follow-up after approximately 12 months and 10 years. We used normative models to compute individual deviation scores for the 2 groups at different timepoints, which allows meaningful comparisons even when follow-up data are acquired on different scanners from the baseline scans.^50^ Then, we assessed the association between CT deviations and symptom scales at clinical follow-up and between deviations and Norwegian patient registry data to address the possibility of selective retention bias influencing our findings. Given prior evidence for the heterogeneity of cortical alterations in schizophrenia^28–30^ and that only a subset of individuals with schizophrenia show progressive brain changes,^36^ we predicted that: (i) we would observe a characteristic yet diffuse pattern of case–control differences in cortical normative deviations, consistent with prior studies^22,24^ and (ii) individual differences in cortical deviations would be coupled to clinical outcome over time. We tested these associations using linear mixed models (LMEs) with subsequent corrections for multiple comparisons.

## Methods

### Participants

All participants were recruited to a specific first-episode sub-study of the TOP study at the University of Oslo and Oslo University Hospital from October 27, 2004, to October 17, 2012. Here, patients with a first-episode schizophrenia spectrum diagnosis (SCZ) were consecutively recruited from the catchment-area-based inpatient and outpatient services at Oslo University Hospital and 3 additional hospitals in the larger Oslo area to the prospective study. Psychiatric diagnosis at baseline was established using the Structured Clinical Interview for DSM-IV Axis I Disorders (SCID-I^40^), and we included a broad range of schizophrenia spectrum diagnoses: schizophrenia (*n* = 57, 72% of the final, quality-checked longitudinal sample), schizophreniform disorders (*n* = 18, 23%), and schizoaffective (*n* = 4, 5%). Information about patients’ current antipsychotic medication was gathered at each timepoint. Positive and negative symptoms were assessed using the Positive and Negative Syndrome Scale (PANSS^51^). Healthy CTRL from the same geographic catchment area were invited based on national records. Exclusion criteria for healthy CTRL included a history of drug or alcohol abuse or dependency, psychosis, bipolar disorder, or major depressive disorder, or having a first-degree relative diagnosed with a psychotic or bipolar disorder. The participants were invited to a follow-up approximately 10 years after their baseline scan (patients mean [SD] 9.7 years [0.9], CTRL 8.2 years [1.1]). A subsample also participated in a follow-up scan after approximately 1 year.^16^ See supplementary figure 2 for details. We also augmented our sample by including additional CTRL from additional Thematically Organized Psychosis (TOP) sub-studies acquired on the same scanners at the same time, to improve the fit of the normative models that we employ.

We also accessed the Norwegian National Registry for healthcare information about all enrolled patients at baseline. This allowed us to access dates and durations of contacts with the Norwegian healthcare system for ICD-10 F-01-09 labeled events (Mental, Behavioral and Neurodevelopmental disorders) in the follow-up period (e.g., from the start of treatment to 10-year follow-up) for all participants, serving as a proxy for illness severity.

### MRI Data Acquisition and Analysis

Three scanners at Oslo University Hospital were used in this longitudinal study without temporal overlap. The first scanner was a 1.5 Tesla Siemens MANETOM Sonata scanner with a 32-channel head coil. T1-weighted images were acquired using an MPRAGE sequence using these parameters: repetition time (TR) = 2.730 ms, echo time (TE) = 3.93 ms, flip angle (FA) = 7°C. The second scanner was a 3 Tesla GE Signa HDxT with an 8HRBRAIN coil. T1-weighted images were acquired using an FSPGR sequence, with the following parameters: TR = 7.8 ms, TE = 3.18 ms, and FA = 12°C. The third scanner was a 3 Tesla GE 750 Discovery scanner with a 32-channel head coil. The T1-weighted images were here acquired using a BRAVO sequence, with the following parameters: TR = 8.16 ms, TE = 3.18 ms, FA = 12°C. See supplementary figure 9 for the distribution of scanners across different timepoints.

T1-weighted structural MRI scans were preprocessed through Freesurfer (version 5.3), and CT measures were parcellated using the Destrieux atlas.^52^ An automatic quality check procedure based on the Freesurfer Euler characteristic was run on all data and samples with a value higher than 5 were removed.^53–58^

### Normative Modeling

To account for site and scanner effects, we used the Hierarchical Bayesian regression (HBR) approach for normative modeling,^54,55,59^ which efficiently accommodates inter-site variation and provides computational scaling, which is useful for multi-cohort and longitudinal studies with data from different scanners. We estimated a normative model for each region of interest (ROI, *n* = 150) in the Freesurfer Destrieux atlas,^52^ using HBR with age as a covariate, and sex and scanner id as batch effects, to predict CT.^45,54,55,58^ This accommodated multi-site pooling using transfer learning and comparisons across scanners.^54,55,59^ The deviations from these models were then used as features in the linear mixed models outlined below. Importantly, as HBR fits site-specific intercepts and slopes, the resulting normative trajectory might not be linear across the lifespan, but rather piecewise linear. Using pooled data from a collection of mostly publicly available datasets from 77 sites, and 40 435 participants,^29^ the reference normative models were first trained on (95%) healthy individuals and validated on an independent set of 2548 CTRL and patients (5%, stratified by sites). We then adapted the model to the 3 unseen Oslo scanners, by transferring the (hyper)parameters as informed priors for these new sites.^55^ For this adaptation step, we used held-out cross-sectional data from CTRL from these 3 scanners, following methods described previously^55^ (supplementary table 1). After this transfer step, we tested the normative models on the remaining longitudinal CTRL and SCZ samples and obtained individual deviation scores for these participants at each timepoint and ROI. We defined the threshold for extreme deviation values as |*z*| > 2.0. While this threshold is arbitrary, we consider 2 standard deviations from the mean a potentially clinically significant effect.

### Statistical Analysis

To test for the potential of nonrandom attrition confounding our findings, we applied *t*-tests to check for differences between the patients followed for 10 years and the ones that dropped out in several factors, i.e., number of hospitalizations, Positive and Negative Syndrome Scale (PANSS) domain scores, and median CT deviation scores.

Next, we employed an LME model to investigate the impact of diagnosis, time since inclusion, age at inclusion, and sex on the deviation scores derived from the 150 cortical ROIs in a longitudinal setting. The interaction between group and time since inclusion (delay) was also included. The model was formulated as follows:


$$\begin{array}{l} y_{i}=\beta_{0}+\beta_{1}X_{1i}+\beta_{2}X_{2i}+\beta_{3}X_{3i}+\beta_{4}X_{4i}+\beta_{5}X_{1i}X_{4i}+u_{i}+\epsilon_{i} \end{array}$$ (1)

where *i* indexes subjects, *y*_*i*_ is the deviation score at a given ROI, and *β*_0_ a global intercept. The variables *X*_1*i*_ , . . ., *X*_4*i*_ represent, respectively, time since inclusion, age at baseline, sex and diagnosis with associated coefficients *β*_1_ , . . ., *β*_4_. In addition, we model an interaction between diagnosis and time since inclusion, i.e., *X*_1*i*_*X*_4*i*_ with coefficient *β*_5_. Finally, *u*_*i*_ is a subject-specific random intercept and $\epsilon_{i}$ are normally distributed errors.

In all instances where multiple comparison correction was required, we applied the Benjamini–Hochberg procedure with α = 0.05 to control the false discovery rate^60^ corrected across ROIs. For the ROIs with a significant interaction effect, we also calculated the predicted values for a combination of time since inclusion (delay) and diagnosis levels to visualize the nature of the interaction effects in the model.

We also aimed to understand the regional distribution of extreme deviations at the level at the individual. As these are count data (i.e., having highly skewed discrete distributions), we applied a non-parametric Wilcoxon test to the proportion of individual deviations differing between diagnostic groups at each timepoint, in line with prior work.^28,29,61^

To examine the development of PANSS scores during the longitudinal period, we visualized the distributions and used an LME model to assess the change in PANSS subscales over time, while controlling for age at baseline and sex. The model was formulated as follows:


$$\begin{array}{l} y_{i}=\beta_{0}+\beta_{1}X_{1i}+\beta_{2}X_{2i}+\beta_{3}X_{3i}+u_{i}+\epsilon_{i} \end{array}$$ (2)

Here, *y*_*i*_ is the PANSS score (or subscale) and *X*_1*i*_, . . . , *X*_3*i*_ are defined as above, respectively, time since inclusion, age at baseline, and sex with coefficients *β*_1_ , . . . , *β*_3_. Again, *β*_0_ is the global intercept, *u*_*i*_ is a subject-specific random intercept and $\epsilon_{i}$ are normally distributed errors.

Next, we employed another LME model to investigate the associations between the deviation score in the different ROIs, time since inclusion, age at inclusion and sex on the general and domain-specific PANSS scores in the schizophrenia patients. The model was formulated as follows:


$$\begin{array}{lll} y_{i}= & \beta_{0}+\beta_{1}X_{1i}+\beta_{2}X_{2i}+\beta_{3}X_{3i} & +\beta_{4}X_{4i}+\beta_{5}X_{1i}X_{4i}+u_{i}+\epsilon_{i} \end{array}$$ (3)

Here, *y*_*i*_, *X*_1*i*_, . . . , *X*_3*i*_, *u*_*i*_, and $\epsilon_{i}$ are defined as in equations (1) and (2), but here *X*_4*i*_ is the deviation score at a given ROI, with coefficient $\beta_{4}$, and we also model an interaction between time since inclusion and deviation score, *X*_1*i*_*X*_4*i*_, with coefficient *β*_5_.

## Results

### Participants

A total of 218 healthy CTRL and 79 patients were included in the longitudinal analysis (table 1 and supplementary figures 1 and 8). There was no significant difference either in the number of contacts with the healthcare system for ICD-10 classified “Mental, Behavioral and Neurodevelopmental disorders,” nor in duration of contacts with the healthcare system (e.g., the duration of treatment) between the longitudinal sample and the drop-outs. There was no significant association between attrition groups and PANSS scores, or median cortical deviation scores. Data on medication use for each timepoint showed that patients primarily used second-generation antipsychotics (62% at baseline, 48% at 1 year, and 17% at 10 years) or did not use any antipsychotics (38%, 50%, and 83%, respectively; for details, see supplementary material).

**Table 1.** Demographics of the Participants at the 3 Time Points

	Baseline		12 months		10 years	
Diagnosis	CTRL	SCZ	CTRL	SCZ	CTRL	SCZ
*N*	218	79	197	67	77	23
Age, mean [SD]	33.9 [10.1]^a^	27.8 [7.6] ^a^	35.1 [10.2] ^a^	29.3 [7.9] ^a^	40.7 [7.8]	37.9 [7.3]
Sex ratio (female)	42.6%	41.7%	40.6%	41.8%	37.6%	34.7%
PANSS, mean [SD]		64 [14]		56 [15]		50 [16]

*Note*: ^a^There was a significant effect of group on age at baseline (*F* = 24.0_1,295_, *P* < .01) and 12 months (*F* = 18.1_1,295_, *P* < .01). There were no other significant differences for other demographic variables.

### Normative Modeling Allows to Compare Across Sites

**Fig. 1.**
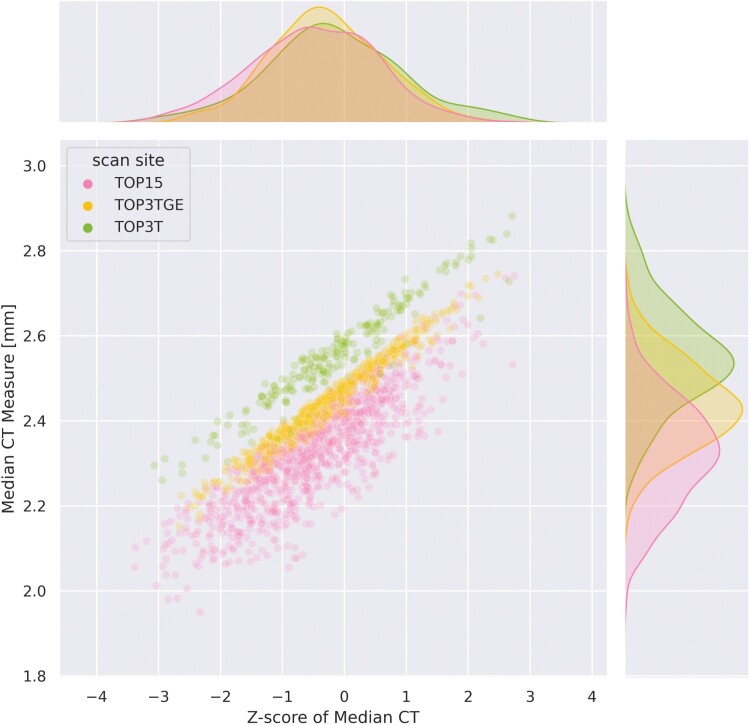
Joint-plot and marginal distributions of the median CT measures (*y*-axis) and associated deviation scores (*x*-axis), color-coded by a scanner, from the test samples of the adaptation set (cross-sectional TOP samples).

Figure 1 displays the joint distributions of the median CT and their associated deviation scores for all healthy CTRL from the validation set of the adaptation (transfer) dataset. Of interest are the marginal densities: normative modeling of the median CT accounting for site and sex aligns the distributions and allows meaningful comparisons between samples from different sites in the deviation scores space. In contrast, estimates of CT appear highly impacted by the site effect (figure 1). We also show an example of the deviation scores following the adaptation process in supplementary figure 8, which illustrates that the normative model does a good job in accounting for age-related variation. We have shown in prior work that normative modeling also allows meaningful comparisons across sex.^40,50,55^

### Deviation Score Difference by ROIs

**Fig. 2.**
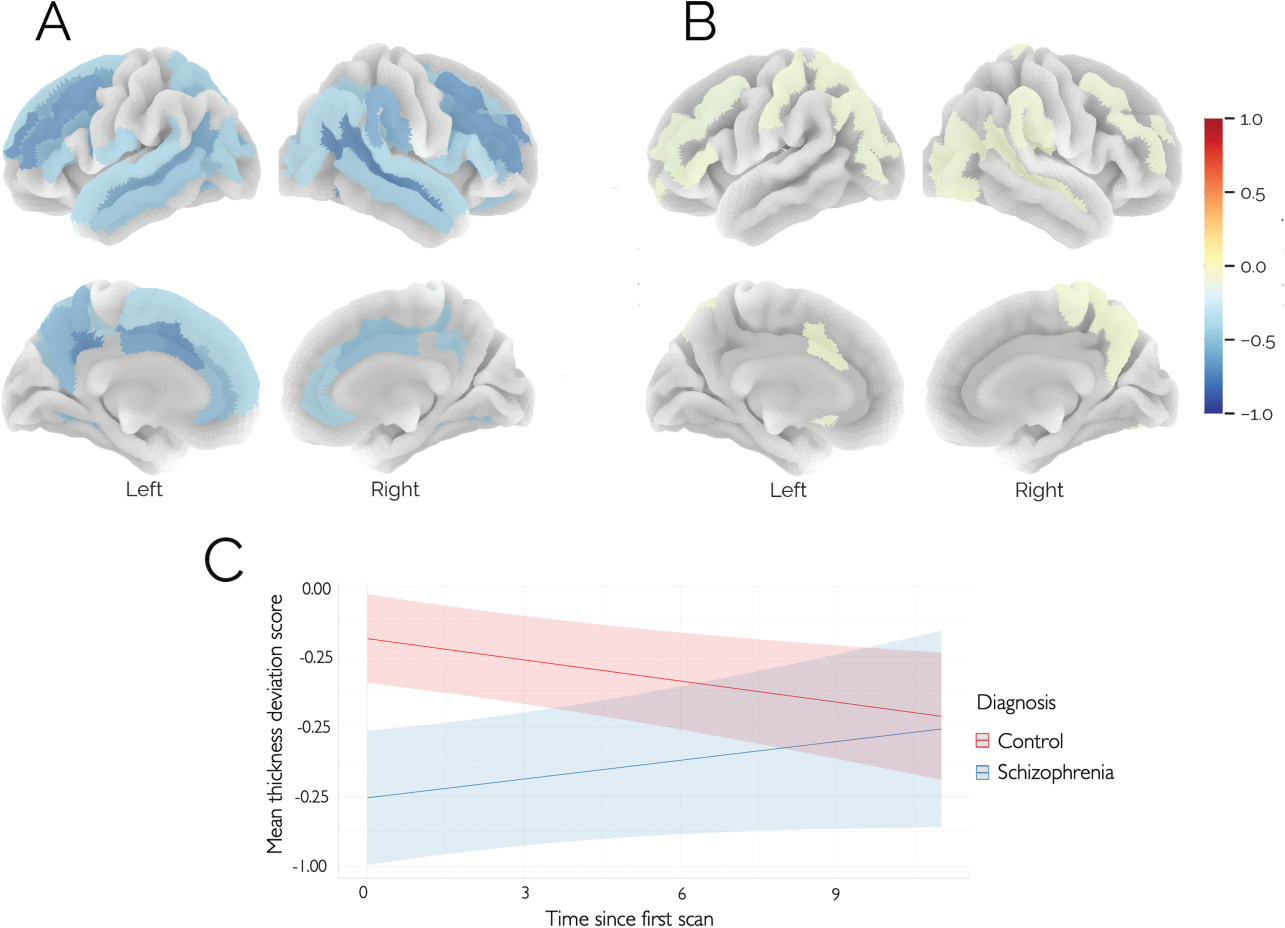
(A) Conditional main effect of diagnosis from the linear mixed model, showing that patients have reduced CT deviations overall (i.e., across all timepoints), color-coded by effect size; (B) time × diagnosis interaction effect, showing ROIs where the effect of diagnosis changes over time; (C) regression plot for mean CT deviation across all ROIs, showing that the differences evident in the deviations at the first timepoint attenuate at later timepoints. The color bar shows the effect size for each effect multiplied by the sign of the coefficient.

LMEs revealed significant (*P* < .05, FDR corrected) conditional main effects of diagnosis and time × diagnosis interaction effects in a diffuse network of lateral temporal, parietal, and frontal brain regions, and along the medial frontal and parietal lobes, bilaterally (figure 2A and B). It should be noted that regression plots for each region (supplementary figure 4) showed a cross-over interaction in many (but not all) regions also having a conditional main effect on diagnosis. In such cases, the interaction effect should be considered the primary finding. Post hoc analyses (supplementary figures 3 and 4) showed that the interaction in most regions was principally due to more negative deviation scores in patients with SCZ at baseline, which attenuate over time such that a fewer number of significant regions were detected at the first follow-up timepoint, and there were no significant differences observed at the final 10-year follow-up (supplementary figures 3–5). The effect sizes at the different timepoints are shown in supplementary figure 3D. Briefly, the Cohen’s *d* for median thickness deviation score at baseline is −0.46, −0.43 at 12-month follow-up and −0.27 at 10-year follow-up. Additionally, there were no significant differences in the deviation scores at baseline between patients who completed the 10-year follow-up scan (*n* = 23) and those who did not but were enrolled at baseline in the 10-year study (*n* = 40). In addition to these effects, we also detected conditional main effects for age, time since inclusion, and sex (supplementary figure 6). However, as these are nuisance effects and all have very small effect sizes, we do not consider them further.

**Fig. 3.**
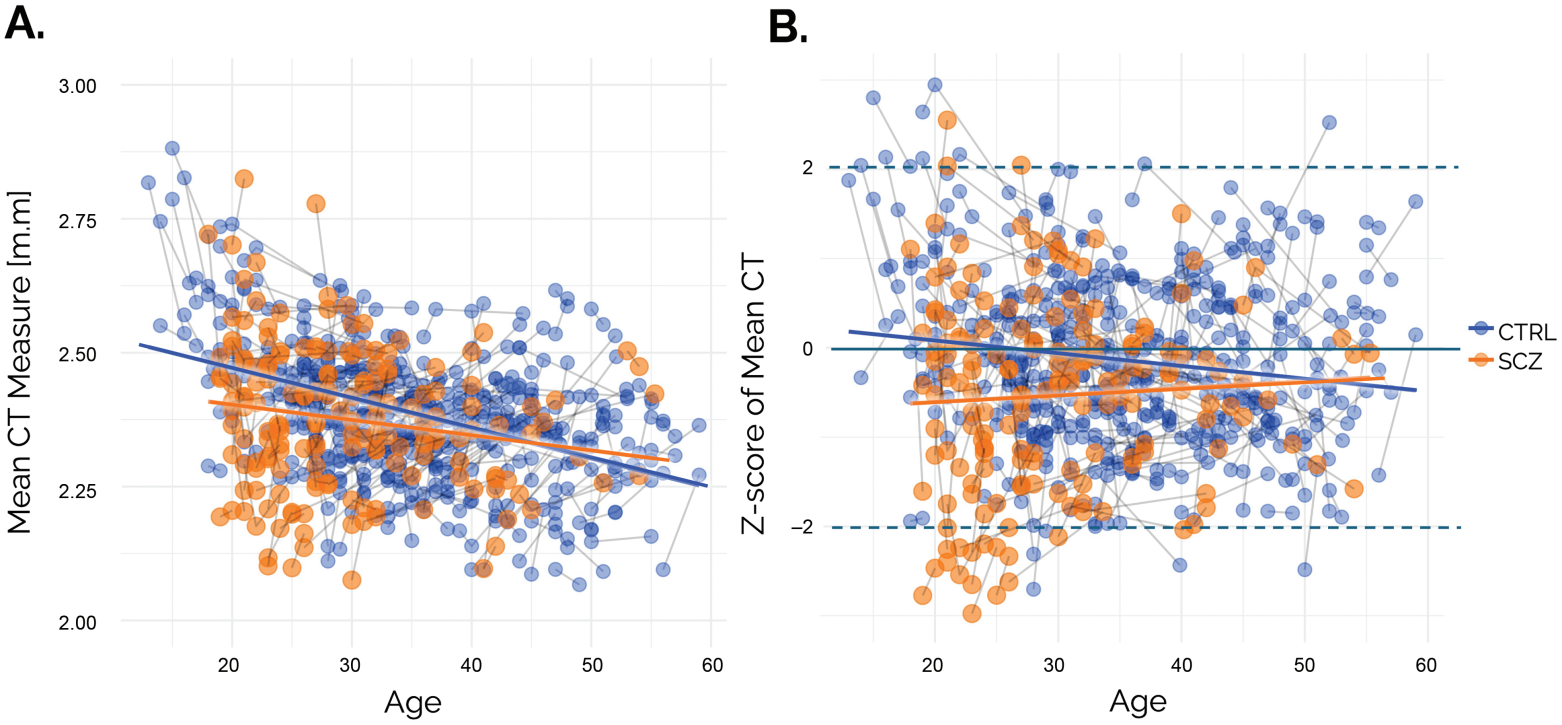
(A) Raw mean CT scores for individuals with schizophrenia and healthy CTRL, where the line segments connect the successive time points of each individual. (B) Deviations from the normative model for mean CT.

For visualization purposes, figure 3 shows both the raw CT estimates and deviations from the normative model for mean CT in individuals with schizophrenia and healthy CTRL. As expected, the raw CT estimates (figure 3A) are confounded by both aging and scanner effects, showing the general reduction in CT that is expected over this lifespan stage.^40^ In contrast, the normative deviations are cleared from these effects (figure 3B). In both cases, the gradual attenuation of baseline reductions in CT over time is apparent.

### Number and Distribution of Regions with Extreme Deviations

**Fig. 4.**
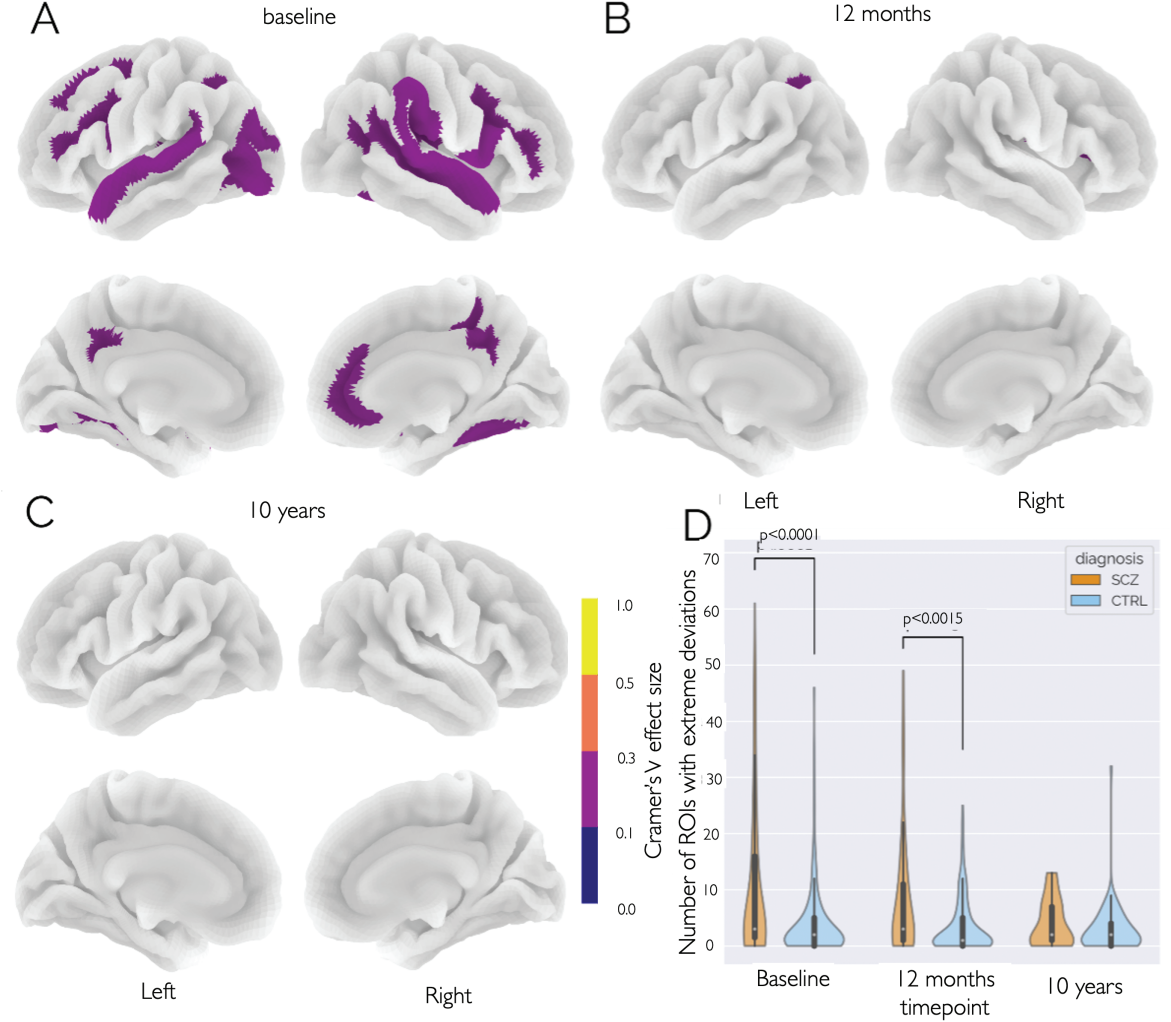
(A–C) ROIs showing a significantly higher proportion of negative extreme deviations among patients with schizophrenia (SCZ) compared with healthy CTRL at each timepoint. (d) *χ*^2^ test significant differences in negative extreme deviation distributions between people with SCZ and CTRL at each time point (see supplementary table 4 for a detailed summary of each ROI).

Figure 4 and supplementary table 3 summarize the distribution of ROIs showing a significantly higher proportion of extreme negative deviations among patients with schizophrenia compared with CTRL, at each timepoint. Here we find ROIs in both the left and right hemispheres with significantly different overlap statistics of extreme negative deviation scores between patients and CTRL. The 3 ROIs with the strongest effects are the lateral aspect of the superior temporal gyrus (CV = 0.19, *P* < .01), and the opercular part of the interferer frontal gyrus (CV = 0.18, *P* < .01) both in the LH, and the superior temporal sulcus (CV = 0.18, *P* < .01) in the right hemisphere. At the second and third time point, none of the ROIs remained significant. A Mann–Whitney *U* test revealed no significant case–control differences in the number of positive extreme deviations, at any time points (*Z* > 2). The analysis revealed significant case–control differences in the number of extreme negative deviations (*Z* < −2) at baseline (*P* = 2.56 × 10^−5^, common language (CL) effect size = 66%) and at the second assessment (*P* = .0006, CL effect size = 63%). There was no significant case–control difference at the third time point (*P* = 1, CL effect size = 50%) (see supplementary tables 4 and 5 for further details).

### Symptom Scores from Inclusion to 10-Year Follow-up

**Fig. 5.**
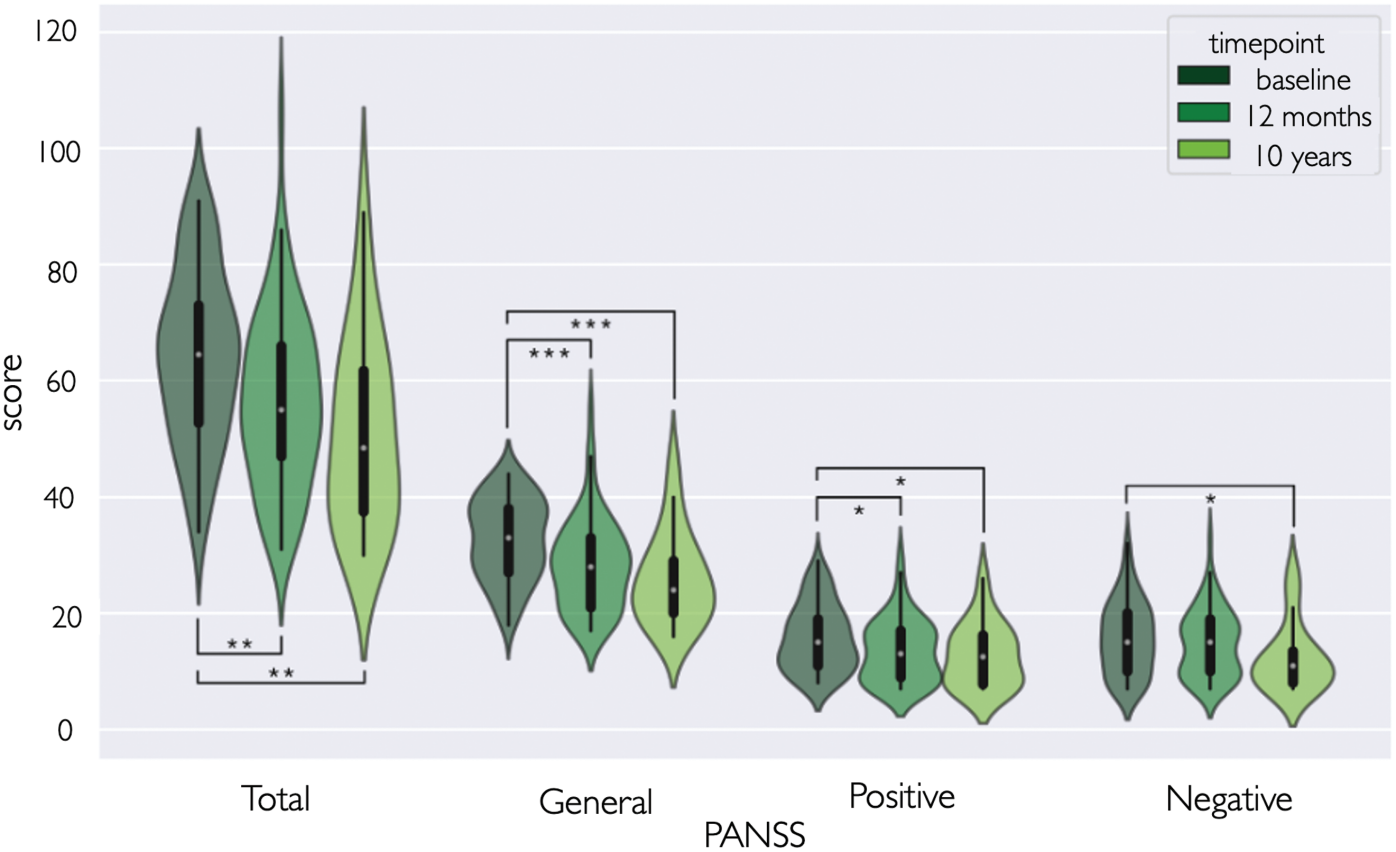
PANSS domain scores at the 3 timepoints. Most follow-up scores are significantly lower than baseline scores indicating a decrease in symptom severity over time (*P < .05, **P < .01, ***P < .001).

PANSS scores at each timepoint are shown in figure 5.

First, linear mixed models were fitted to examine the association between the different PANSS sub-scores and the predictor variables, including time since the first scan, age at the first scan, and sex. For all PANSS scores, there was a significant effect of time (*P* < .001), indicating that PANSS scores decreased over time. None of the other covariates were significant (supplementary figure 10). To further clarify the nature of these effects, we also tested differences between individual timepoints, significant differences were observed only when comparing baseline values to the second follow-up scores, but not between the 2 follow-up time points, indicating that the main improvement took place during the first year (baseline—12-month follow-up, *P* = .002 Cohen’s *d* = −0.53; baseline—10-year follow-up *P* = .001, Cohen’s *d* = −0.87). For PANSS subscales, we notice significantly lower scores for the general psychopathology scale at follow-ups compared with baseline (baseline—12-month follow-up *P* = .0002 Cohen’s *d* = −.60; baseline—10-year follow-up *P* = .0004 Cohen’s *d* = −0.93). Symptom domains were also significantly reduced at 12-month and 10-year follow-ups compared with baseline for positive symptoms (baseline—12-month follow-up *P* = .012 Cohen’s *d* = −0.42; baseline—10-year follow-up *P* = .026 Cohen’s *d* = −.56) and at 10 years for negative symptoms (baseline—10-year follow-up *P* = .019 Cohen’s *d* = −0.58).

### Association of CT Deviation and PANSS Scores

**Fig. 6.**
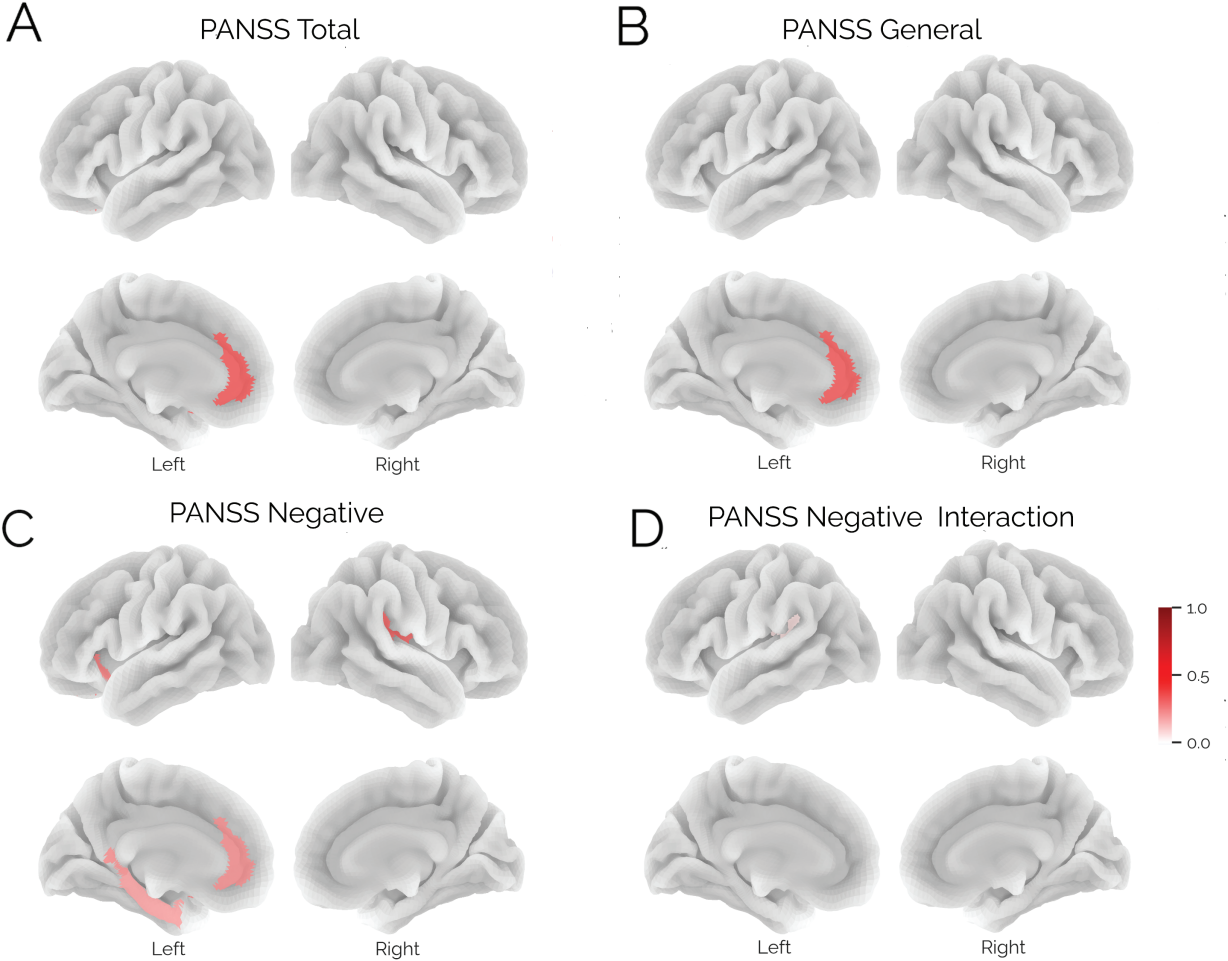
Results from the LME model testing for associations between symptom scores and cortical deviations (equation 2). We report man effects for the PANSS total domain (A), the PANSS general domain (B), PANSS negative symptoms (C), and a significant interaction in a single region between the PANSS negative scores and follow-up time (D). The color bar shows the effects size of each effect.

Figure 6 shows the results from the LME testing for associations between cortical deviations and PANSS. Several ROIs in the LH had a significant association with PANSS domain scores across time. Anterior cingulate gyrus was associated with PANSS total and PANSS general (coefficient = −4.0 and −1.79, respectively, *P* = .003 and .01). The left anterior segment of the circular sulcus of the insula (coefficient = −1.3, *P* = .04), the posterior ramus of the lateral sulcus (coefficient = 1.52, *P* = .03), and the medial orbital sulcus (coefficient = −1.42, *P* = .02) were also significantly associated with PANSS negative. Negative associations indicate more negative deviation scores with higher symptom severity.

There was a significant interaction between time since inclusion and the LH posterior ramus (or segment) of the lateral sulcus on PANSS negative scores (figure 5D, coefficient = 0.28, *P* = .047), indicating that an initial association between larger negative deviations and more symptoms at baseline attenuated with time (see supplementary figure 6).

## Discussion

In this study, we analyzed CT data from a 10-year longitudinal study of people with schizophrenia and healthy CTRL using structural brain MRI. The dataset included 2 or 3 time points for each participant, covering a period of approximately 10 years. We used normative modeling to investigate the deviations from an expected pattern of CT and how this pattern changed over time, both at the group level and at the individual level. We also examined the relationship between these deviation scores and the severity of psychotic symptoms as measured by PANSS. We report 3 main findings: (i) we show a diffuse pattern of CT atypicalities in schizophrenia early in the illness course, both in terms of the mean deviations across groups and in the number of extreme deviations at the individual level; and that these deviations (ii) attenuate over time; and (iii) associations with clinical symptoms across a distributed set of brain regions.

The pattern of negative deviation scores at baseline in a diffuse network of in patients compared with CTRL encompassed bilateral temporal, parietal and frontal regions. These significant effects are consistent with findings from large meta-analytic studies^22–24^ and more generally,^14,28,62^ present both at baseline and at the first follow-up and are evident at the group and individual levels, both in terms of the mean and the number of extreme deviations (figures 2 and 4, respectively). Significant interactions between diagnosis and time demonstrate that these differences attenuate over time. More specifically, between the first two time points, the number of ROIs with significant effects decreased, but also the amplitude of the remaining effects. These findings are in line with previous reports that the gray matter differences were most severe in the early years after schizophrenia onset,^36^ and are discordant with a general notion of schizophrenia as a neurodegenerative disorder with progressive brain aberrations over time.^25,36^ The 3 previous long-term prospective brain imaging studies of first-episode psychosis also found that only a smaller subset of individuals with schizophrenia showed significant progressive brain changes and also had a high proportion of subjects using first-generation antipsychotics.^36,37,39^ In contrast, in our study, individuals with psychosis were almost exclusively treated with second-generation antipsychotics. In view of the heterogeneity within the illness^28–30^ and since the proportion of patients with stable, poor clinical trajectories is relatively low, and the proportion with increasingly severe trajectories is even lower, we consider that the profile we detect likely reflects that very few participants with these trajectories are part of our study sample. Our findings thus primarily reflect the more common favorable trajectories. Since good-outcome first-episode patients leave the treatment services, more extensive cross-sectional studies based on clinical recruitment will include more multi-episode patients. We consider that cross-sectional studies will thus be enriched with patients with more severe trajectories, explaining the findings of considerable heterogeneity found in these types of studies.^28,29,63^

The pattern of brain regions showing case–control differences particularly implicated frontal and temporal regions including the paracentral lobule and sulcus, which have been associated with poor 1-year functional outcomes^64^ and the superior temporal gyrus, associated with positive symptoms.^62^ Several insula ROIs showed more negative deviations in participants with schizophrenia than in CTRL at both baseline and at the first follow-up after on average 24 months. This region, especially on the left, is associated with inner speech and verbal hallucinations and reduced insula gray matter has been reported in hallucinators.^65^

We assessed the possibility of nonrandom attrition biasing our findings, which is often a concern in longitudinal studies and may influence the validity of regression models.^66^ Logistic regressions and LME models did not indicate any significant differences between attrition and CT deviation scores, PANSS scores, or in the frequency or duration of contacts with the healthcare system for mental, behavioral, and neurodevelopmental disorder-related events.

Linear mixed models revealed a few brain regions showing associations with symptoms. Most notably in the anterior cingulate gyrus where patients with more negative deviations exhibited higher symptom scores on multiple PANSS domains (total, positive, and negative scales) and several other regions including the insula and parahippocampal gyrus showed an association with negative symptoms. Notably, alterations in the insula and the cingulate gyrus have been associated with negative symptoms, hallucinations, and psychotic disorders.^22,67,68^

The observed case–control differences in CT deviation scores at baseline, reflecting a relatively early clinical phase, suggest that brain differences might be observable before the onset of the first episode. Indeed, large meta- of mega-analytic studies have shown that cortical alterations are present in the at-risk phase,^69^ although it has also been shown that such changes explain only a tiny proportion of the variance in regional deviations from a normative model for CT and do not predict conversion to psychosis.^70^ In individuals at clinical high risk for psychosis, multimodal (including brain MRI data) prediction of the negative symptom severity appears to yield promising results.^71^ The notion of a neurodevelopmental component in the etiology of severe mental disorders is in line with previously reported correlations between deviation in CT and a general psychopathology score in a population-based sample of children and adolescents.^72^ However, we emphasize that our data cannot inform directly about the neurodevelopmental antecedents of schizophrenia because we lack information from important neurodevelopmental phases.^41^

## Limitations

Our study is subject to several limitations. First, even with access to the national registry data, ensuring representative recruitment in clinical and population-based cohorts is nontrivial, e.g., inclusion and exclusion criteria of patients^73,74^ or bias on the selection of healthy subjects^75^ or the retention of individuals with psychosis and CTRL. We are currently working on an extensive evaluation of these potential biases in normative models in separate work.

Second, whilst our findings are suggestive that second-generation antipsychotics may have different chronic effects on brain structure to first-generation antipsychotics, we were unable to test this directly because only a very small number of participants were taking first-generation antipsychotics in our sample. Further work is therefore necessary to test this more directly.

Third, extreme cortical deviations may not only relate to schizophrenia-related pathologies but could also be markers of other effects, e.g., noise, artifacts, medications, co-morbidities, co-existing conditions, and various lifestyle and health-related behaviors or traits.^76^ While we cannot rule out confounding effects, our quality control and validation procedures against clinical and registry data speak against this interpretation.

Finally, we acknowledge that our sample size at the third follow-up session is moderate, and this reduction in sample size could have biased our findings at later timepoints. However, we would like to emphasize that: (i) the proportion of subjects retained in our study compares favorably to the retention rates reported in the literature, particularly in view of the 10-year follow-up period of this study and (ii) the effect size estimates we present (supplementary figures 3 and 4) also speak against the possibility that the attenuation of effects we report is only attributable to a reduction in sample size. Also, despite best efforts, the inclusion of different scanners across different waves of the study may have influenced our findings. However, we have extensively validated the normative modeling framework that we employ in such settings elsewhere and it shows good performance.^50^ Nevertheless, our findings should be considered preliminary at this stage and await replication in other cohorts.

## Conclusion

Using a unique dataset comprising clinical and MRI data from a 10-year longitudinal study with patients with schizophrenia, we have shown an apparent gradual reduction in case–control CT deviations from the first psychotic episode to the 10-year follow-up assessment, with some evidence of regionally distributed associations with clinical symptoms over time. This study demonstrates that transfer learning from large-scale reference normative models can be used to make meaningful comparisons of MRI features between participants across different scanners and provides preliminary evidence for cortical associations with longitudinal clinical outcomes in people with schizophrenia.

## Supplementary Material

Supplementary material is available at https://academic.oup.com/schizophreniabulletin/.

sbae107_suppl_Supplementary_Figures_1-9_Tables_1-5
